# The impact of proactive personality on career choice anxiety among university students: the chain mediating roles of intolerance of uncertainty and career decision making difficulties

**DOI:** 10.3389/fpsyg.2026.1756689

**Published:** 2026-03-18

**Authors:** Na Li, Jinlou Xie, Zhengchun Guo, Yinnan Yang

**Affiliations:** School of Economics and Management, Changzhou Institute of Technology, Changzhou, Jiangsu, China

**Keywords:** career choice anxiety, career decision-making difficulties, chain mediation, intolerance of uncertainty, proactive personality

## Abstract

**Objective:**

Based on the theoretical frameworks of social cognitive career theory and cognitive-behavioral theory, this study aimed to construct a chain mediation model to explore the impact of proactive personality on career choice anxiety among university students, with a specific focus on the mediating mechanisms of intolerance of uncertainty and career decision-making difficulties.

**Methods:**

A convenience sampling method was employed, and 555 university students from Jiangsu Province were surveyed using questionnaires. The measurement tools included the Proactive Personality Scale, Intolerance of Uncertainty Scale, Career Decision-Making Difficulties Scale, and Career Choice Anxiety Scale. Structural equation modeling was conducted to assess the model fit, and the bias-corrected percentile Bootstrap method was used to test the mediation effects, evaluating the significance of direct, indirect, and chain mediation effects.

**Results:**

(1) A significant negative correlation was observed between proactive personality and career choice anxiety (*β* = −0.173, *p* < 0.01).(2) Intolerance of uncertainty was found to partially mediate the relationship between proactive personality and career choice anxiety (effect = −0.099, 95% CI [−0.1375, −0.0637]).(3) Career decision-making difficulties were identified as a partial mediator between proactive personality and career choice anxiety (effect = −0.062, 95% CI [−0.0904, −0.0355]).(4) A significant Chain mediation effect was demonstrated through intolerance of uncertainty and career decision-making difficulties in the association between proactive personality and career choice anxiety (effect = −0.034, 95% CI [−0.0486, −0.0208]). This chain mediation pathway reveals an underlying mechanism progressing from “cognitive appraisal” to “behavioral decision-making” and ultimately to “emotional outcomes.”

**Conclusion:**

Proactive personality was not only directly and negatively associated with career choice anxiety but also showed indirect links to it through the independent mediating roles of intolerance of uncertainty and career decision-making difficulties, as well as their chain mediation pathway. From a cognitive-behavioral perspective, this study elucidates the formation mechanism of career choice anxiety, providing a theoretical basis and practical implications for career psychological interventions in higher education.

## 1 Introduction

Against the macro-backdrop of profound transformations in the global economic structure and the ongoing optimization of China’s industrial structure, the employment environment for university students is characterized by notable complexity and uncertainty (Xiang et al., 2023). According to statistics from the Ministry of Education, the number of graduates from Chinese higher education institutions in 2023 reached a record high of 11.58 million. However, the absorptive capacity of the job market has not increased correspondingly, leading to intensified employment competition (Lian, 2023). Concurrently, the rapid emergence of new industries and the digital transformation of traditional sectors have resulted in increasingly diversified and blurred career pathways. The explosion of occupational information and the growing uncertainty regarding professional boundaries further exacerbate the career selection pressure faced by university students (Gedrimiene et al., 2024). Within this context, Career choice anxiety has become a prevalent psychological phenomenon among the university student population, manifested as excessive worry about employment prospects, lack of confidence in one’s own abilities, and avoidance or procrastination in the career decision-making process (Taşkıran et al., 2025). If such a state of anxiety persists over the long term, it can not only impair an individual’s mental health but also adversely affect the quality and efficiency of their career decisions, thereby hindering long-term career development (Fang et al., 2025). Consequently, a thorough investigation into the psychological mechanisms of career choice anxiety among university students and the identification of effective intervention pathways have become crucial issues that need to be addressed urgently in the fields of educational psychology and career counseling.

Among the various factors influencing career choice anxiety in university students, individual psychological characteristics—particularly personality traits—have garnered increasing research attention. Proactive personality, as a stable behavioral tendency, is characterized by the disposition to take initiative to alter one’s environment, actively seek out opportunities, and persistently pursue goals. It is regarded as a significant psychological resource for coping with career challenges (Li et al., 2014). Individuals with a highly proactive personality are generally found to be more capable of formulating clear career plans, expanding social support networks, and employing proactive coping strategies when confronted with job-seeking pressures, thereby potentially alleviating anxiety during the career selection process (Li et al., 2014). However, existing studies have primarily focused on examining the direct effects of proactive personality on employment behaviors or outcomes (Liu and Shi, 2023), whereas its underlying pathways within the formation of career choice anxiety have not been sufficiently elucidated. Particularly in the current highly uncertain employment environment, individuals’ cognitive and emotional responses to ambiguous situations may serve as critical variables affecting their psychological adaptation.

In summary, this study is designed to establish a Chain mediation model to systematically examine the mediating roles of intolerance of uncertainty and career decision-making difficulties in the relationship between proactive personality and career choice anxiety among university students. By integrating multi-level variables encompassing personality traits, cognitive biases, and decision-making behaviors, the research aims to elucidate the underlying psychological pathways through which proactive personality influences career choice anxiety. The findings are expected to provide theoretical insights for a deeper understanding of the multi-faceted mechanisms underlying career choice anxiety, along with empirical evidence to inform career psychological counseling and intervention practices in higher education.

### 1.1 Proactive personality and career choice anxiety among university students

Proactive personality is defined as a stable individual trait characterized by a behavioral tendency to take initiative and act to change external environments, irrespective of situational constraints (Chai et al., 2022; Seibert et al., 1999). Within the framework of Social Cognitive Career Theory (SCCT), personality traits are regarded as important antecedent variables in the career development process, influencing emotional outcomes through their effects on cognitive and behavioral patterns (Schaub and Tokar, 2005). University students with a highly proactive personality are more inclined to adopt strategies such as active exploration, planning, and proactive social networking when confronted with career-related challenges (Wang et al., 2021). These behaviors are considered to effectively enhance their sense of control over the career world and strengthen self-efficacy, thereby serving as important psychological resources for coping with career selection stress (Valls et al., 2020). Substantial empirical evidence has demonstrated that proactive personality is positively correlated with adaptive variables such as career decision-making self-efficacy and career adaptability, while being negatively correlated with negative emotional states such as anxiety and depression (Li et al., 2025). Consequently, it is proposed in this study that a higher level of proactive personality is associated with lower levels of anxiety experienced during the career decision-making process. Based on this reasoning, the following hypothesis is proposed: *H1*: Proactive personality is significantly and negatively associated with career choice anxiety among university students.

### 1.2 The mediating role of intolerance of uncertainty

Intolerance of Uncertainty (IU) is defined as a dispositional tendency to respond negatively to ambiguous, uncertain, or unpredictable situations, accompanied by adverse cognitive, emotional, and behavioral reactions (Zhang et al., 2023). According to cognitive mediation theory, the influence of personality traits on emotional outcomes is often realized through cognitive appraisal processes. In the context of career decision-making, proactive personality may shape an individual’s cognitive appraisal of uncertain situations, which in turn affects their emotional responses such as anxiety. In the current complex and volatile employment environment, uncertainty has become apersistent condition that university students must confront (Shdaifat et al., 2024). Individuals with a proactive personality are considered adept at seizing opportunities and actively altering their environment; this trait is associated with a higher tolerance for uncertainty and a greater tendency to perceive uncertainty as a challenge rather than a threat. In contrast, individuals with a low level of proactive personality may experience more intense discomfort and worry about future uncertainties due to a lack of proactive coping strategies (Sacchi and Dan-Glauser, 2024). Specifically, individuals with a high level of proactive personality tend to appraise uncertainty as a challenge rather than a threat, demonstrating greater cognitive flexibility and a higher tolerance for ambiguity. This positive cognitive appraisal reduces intolerance of uncertainty, thereby mitigating subsequent anxiety. In contrast, individuals with low proactive personality are more likely to exhibit rigid and negative appraisals of uncertainty, leading to higher intolerance of uncertainty and, consequently, elevated career choice anxiety. Empirical evidence indicates that intolerance of uncertainty serves as a core cognitive variable in anxiety disorders and is a significant positive predictor of anxiety levels (Buhr and Dugas, 2006). Therefore, it is proposed that proactive personality may alleviate career choice anxiety by reducing an individual’s level of intolerance of uncertainty. Based on this rationale, the following hypothesis is formulated: *H2*: Intolerance of uncertainty mediates the relationship between proactive personality and career choice anxiety among university students.

### 1.3 The mediating role of career decision making difficulties

Career decision making difficulties refer to a state in which individuals experience challenges in making career choices due to inadequate preparation or various encountered obstacles. According to career construction theory, successful adaptation to the vocational environment requires active career behaviors (e.g., exploration, planning), which not only reduce decision-making difficulties but also enhance psychological adjustment (Talluri et al., 2024). University students with a highly proactive personality are typically engaged in active career exploration, information gathering, and clarification of self-concept and career goals. These behaviors are considered effective in mitigating difficulties arising from insufficient information, ambiguous goals, or internal conflicts during the decision-making process (Yu et al., 2021). Furthermore, career decision-making difficulties have been empirically established as a significant proximal factor triggering career choice anxiety. When individuals perceive decision-making as challenging or are filled with doubts about potential outcomes, anxiety levels are observed to increase significantly (Shi, 2023). Therefore, it is proposed that proactive personality may indirectly reduce career choice anxiety by facilitating active career exploration and planning behaviors, thereby decreasing career decision-making difficulties. Based on this rationale, the following hypothesis is proposed: *H3*: Career decision-making difficulties mediate the relationship between proactive personality and career choice anxiety among university students.

### 1.4 The chain mediating roles of intolerance of uncertainty and career decision-making difficulties

Cognitive-behavioral theoretical models posit that an individual’s cognitive appraisal directly influences subsequent behavioral performance and emotional outcomes. In the domain of career decision-making, cognitive tolerance for uncertainty is recognized as a fundamental cognitive basis for decision-making behaviors (Lee and Jung, 2021). Specifically, individuals characterized by high intolerance of uncertainty tend to experience excessive worry and develop avoidance tendencies due to the unpredictability of future career outcomes. This cognitive-emotional response is observed to undermine their willingness and capacity to engage in thorough career exploration and make decisive choices, thereby exacerbating career decision-making difficulties (Storme and Celik, 2018). This suggests that proactive personality may initially moderate an individual’s cognitive response to uncertainty, which in turn is expected to facilitate more effective career decision-making behaviors, ultimately leading to a reduction in career choice anxiety (He et al., 2021). This sequential pathway—from cognition to behavior, and then to emotion—provides a more systematic and comprehensive framework for elucidating the underlying psychological mechanism through which proactive personality influences career choice anxiety. Based on this theoretical reasoning, the following hypothesis is proposed: *H4*: Intolerance of uncertainty and career decision-making difficulties have a chain mediating effect between proactive personality and career choice anxiety among university students.

**Figure 1:**
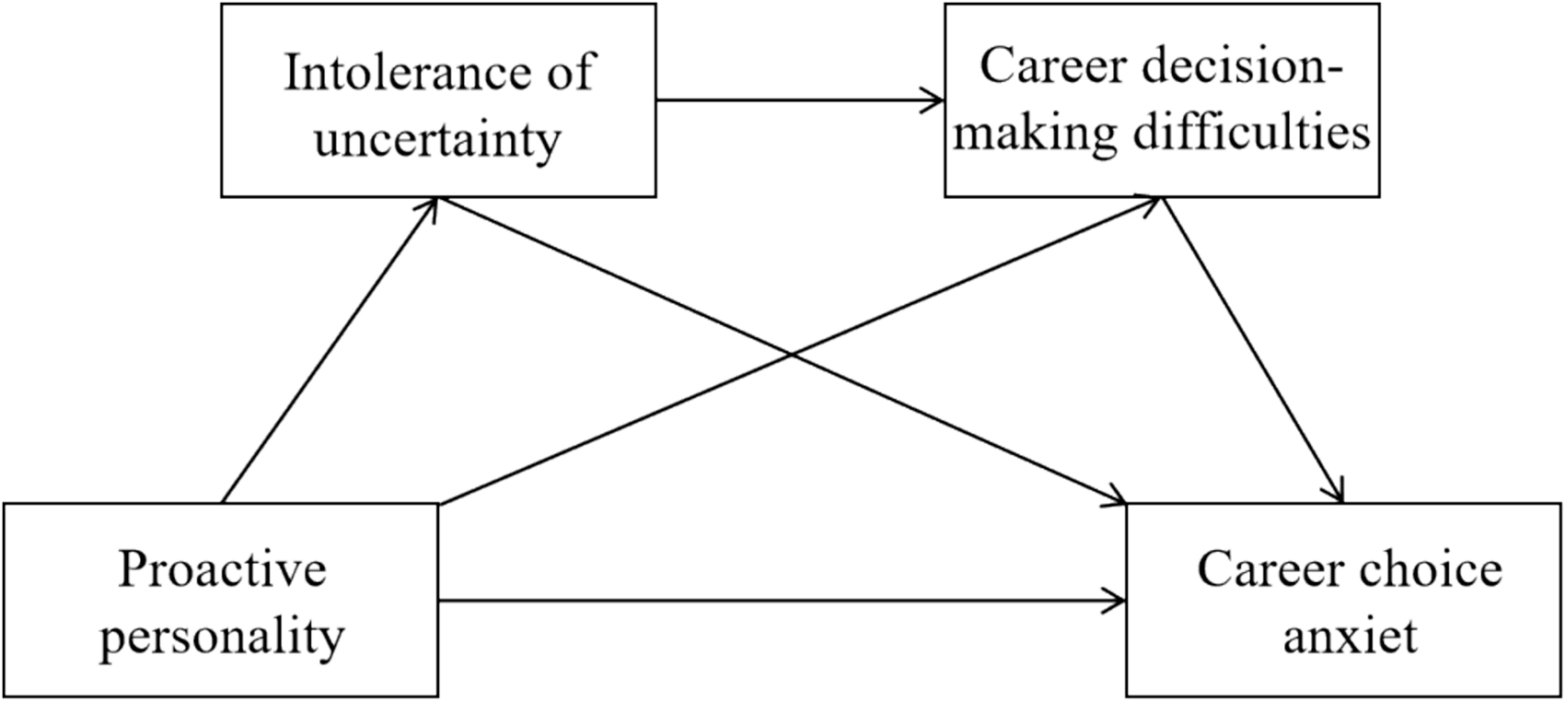
Research model of the impact of proactive personality on career choice anxiety among university students.

In summary, intolerance of uncertainty and career decision-making difficulties are introduced in this study, through which a Chain mediation hypothesis model (as shown in Figure 1) is constructed between proactive personality and career choice anxiety among university students, in order to examine the mechanism underlying this influence.

## 2 Methods

### 2.1 Participants

A cross-sectional survey design was employed in this study, with university students from institutions in Jiangsu Province, China, serving as the target population. Participants were recruited using a convenience sampling method through the online platform “Questionnaire Star.” A total of 598 questionnaires were collected. Following data cleaning, 43 invalid responses—including those with inconsistent answers to reverse-scored items and patterned responses—were excluded, resulting in 555 valid questionnaires and an effective response rate of 92.81%. Among the valid sample, 379 participants (68.3%) were male and 176 (31.7%) were female. The distribution by academic year was as follows: 194 freshmen (34.9%), 172 sophomores (31.0%), 122 juniors (22.0%), and 67 seniors (12.1%). All participants were informed of the research purpose and voluntarily took part after providing informed consent. The study procedures were strictly conducted in accordance with ethical standards, with anonymity and confidentiality of the data ensured throughout.

### 2.2 Measures

#### 2.2.1 Proactive personality

The Proactive Personality Questionnaire (PPQ), revised by Seibert et al. (1999), was used for measurement. This scale consists of 10 items. Responses were recorded on a 7-point Likert scale (1 = “strongly disagree” to 7 = “strongly agree”). A total score was calculated by summing the scores of all items, with higher total scores indicating a stronger proactive personality trait. In this study, the Cronbach’s *α* coefficient for the questionnaire was found to be 0.854.

#### 2.2.2 Career choice anxiety

The Career Choice Anxiety (CCA) scale developed by Zhang and Chen (2006) was utilized. This scale comprises 26 items across four dimensions: lack of self-confidence, employment competition pressure, lack of employment support, and concerns about employment prospects. Responses were measured using a 5-point Likert scale, where higher scores indicate higher levels of career choice anxiety. In the present study, the Cronbach’s *α* coefficient for this scale was determined to be 0.838.

#### 2.2.3 Intolerance of uncertainty

The Intolerance of Uncertainty Scale (IUS-12), revised by Carleton et al. (2007), was administered. This scale is divided into two dimensions and consists of 12 items. A 5-point Likert scale was employed, ranging from 1 (“strongly disagree”) to 5 (“strongly agree”). Higher scores indicate lower tolerance for uncertainty, reflecting a more severe tendency toward intolerance of uncertainty. In this study, the scale was found to have a Cronbach’s *α* coefficient of 0.860.

#### 2.2.4 Career decision making difficulties

The Career Decision Making Difficulties Questionnaire (CDDQ) compiled by Du and Long (2006) was administered. This scale consists of 16 items across four dimensions: career information, career self, career planning, and career goal determination. A 5-point Likert scale was utilized, ranging from 1 (“strongly disagree”) to 5 (“strongly agree”). Higher scores indicate fewer career decision-making difficulties, while lower scores reflect more severe difficulties in this domain. In the present study, the Cronbach’s *α* coefficient for this scale was determined to be 0.833.

### 2.3 Data analysis

Data analysis was performed using SPSS 26.0 and AMOS 28.0 software. First, Harman’s single-factor test was conducted to assess common method bias. Second, descriptive statistics were employed to analyze demographic characteristics, and Cronbach’s α coefficients were calculated to evaluate the reliability of the scales. Subsequently, Pearson correlation analysis was utilized to examine the relationships among proactive personality, intolerance of uncertainty, career decision-making difficulties, and career choice anxiety. Finally, the Chain mediation effects involving proactive personality, intolerance of uncertainty, career decision-making difficulties, and career choice anxiety were tested using PROCESS (Model 6).

## 3 Results

### 3.1 Common method bias test

As all research data were collected through questionnaires, potential common method bias was addressed. During data collection, measures including anonymous responses and mixed positive/negative scoring were implemented for control. Furthermore, Harman’s single-factor test was employed for validation. The unrotated factor analysis revealed four factors with eigenvalues greater than 1, with the first factor accounting for 36.345% of the variance, which is below the critical threshold of 40% (Podsakoff et al., 2003). These results indicate that no serious common method bias was present in this study.

### 3.2 Confirmatory factor analysis

First, the measurement model was evaluated, and the results indicated that all fit indices fell within acceptable thresholds, with the model demonstrating excellent overall fit. The exceptionally low RMSEA observed may be attributed to the moderate sample size, the clarity of the model structure, and the use of well-validated measurement instruments with established reliability and validity. Although such a low RMSEA value is relatively uncommon in empirical research, when considered in conjunction with other fit indices (e.g., χ^2^/df), the measurement model was found to exhibit strong robustness and explanatory power. Subsequently, a confirmatory factor analysis was conducted to comprehensively assess the reliability and validity of the measurement model. Key metrics, including standardized factor loadings, average variance extracted (AVE), and composite reliability (CR), were calculated (Uzun et al., 2025). As shown in Table 1, all factor loadings and AVE values exceeded 0.50, indicating satisfactory convergent validity. Furthermore, both CR values and Cronbach’s *α* coefficients were above 0.70, supporting strong discriminant validity. Additionally, all standardized factor loadings were greater than 0.65, AVE values exceeded 0.50, and CR values were above 0.80, which further corroborated the convergent and discriminant validity of the measurement model. Therefore, it is concluded that the measurement model is statistically reliable and valid.

**Table 1:** The validity and reliability values of the measurement model.

Variable	Observational variable	Unstd	S. E.	C. R.	*p*	Std	CR	AVE
Proactive personality	PP1	1				0.771	0.855	0.542
	PP2	0.961	0.06	16.14	***	0.706		
	PP3	1.013	0.057	17.782	***	0.776		
	PP4	0.903	0.057	15.723	***	0.689		
	PP5	0.952	0.056	16.866	***	0.736		
Intolerance of uncertainty	IOU1	1				0.801	0.839	0.566
	IOU2	0.906	0.054	16.735	***	0.713		
	IOU3	0.897	0.053	16.961	***	0.722		
	IOU4	0.983	0.054	18.152	***	0.770		
Career decision-making difficulties	CDD1	1				0.787	0.861	0.554
	CDD2	0.934	0.054	17.429	***	0.734		
	CDD3	0.969	0.054	17.907	***	0.752		
	CDD4	0.922	0.056	16.505	***	0.699		
	CDD5	0.903	0.051	17.768	***	0.747		
Career choice anxiety	CCA1	1				0.780	0.835	0.560
	CCA2	0.892	0.056	16.001	***	0.691		
	CCA3	1.046	0.056	18.758	***	0.805		
	CCA4	0.95	0.058	16.502	***	0.711		

The symbol *** presented in Table 1 indicates that the *p*-value is less than 0.001 (*p* < 0.001), meaning that the results are highly statistically significant.

### 3.3 Descriptive statistics and correlations

Descriptive statistics and correlation analyses were conducted for the four main study variables, with detailed numerical results presented in Table 2. The results indicated that the mean values of these variables ranged between 3.000 and 3.015, while correlation coefficients ranged from 0.251 to 0.564, all of which were statistically significant at the 0.01 level. Specifically, significant negative correlations were observed between intolerance of uncertainty, career decision-making difficulties, career choice anxiety, and proactive personality. In contrast, significant positive correlations were found between career decision-making difficulties, career choice anxiety, and intolerance of uncertainty. A significant positive correlation was also identified between career choice anxiety and career decision-making difficulties. These correlation patterns were consistent with the research hypotheses.

**Table 2:** Descriptive statistics and correlations.

Variable	M	SD	Proactive personality	Intolerance of uncertainty	Career decision-making difficulties	Career choice anxiety
Proactive personality	3.013	0.792	1			
Intolerance of uncertainty	3.000	0.839	−0.251**	1		
Career decision-making difficulties	3.001	0.813	−0.288**	0.451**	1	
Career choice anxiety	3.015	0.827	−0.356**	0.564**	0.537**	1

**p < 0.01, M, mean, SD standard deviation.

### 3.4 Chain mediating effect test

Path analysis was conducted on the research model, with all path coefficients reaching statistical significance, as detailed in Table 3. The data analysis revealed that proactive personality was significantly and negatively associated with intolerance of uncertainty (*β* = −0.301, *p* < 0.01). In subsequent pathways, intolerance of uncertainty was not only observed to significantly and positively predict career decision-making difficulties (*β* = 0.471, *p* < 0.01), but also demonstrated a significant positive predictive effect on career choice anxiety (*β* = 0.443, *p* < 0.01), thus supporting Hypothesis 2. Concurrently, proactive personality showed a significant direct negative association with career decision-making difficulties (*β* = −0.188, *p* < 0.01), while career decision-making difficulties were shown to significantly and positively influence career choice anxiety (*β* = 0.342, *p* < 0.01), thereby confirming Hypothesis 3. Additionally, a significant direct negative association between proactive personality and career choice anxiety was also identified (*β* = −0.173, *p* < 0.01), supporting Hypothesis 1. Regarding the model’s explanatory power, the variance explained for career decision-making difficulties was calculated at 31.2%, while that for career choice anxiety reached 58.9%, indicating strong predictive efficacy of the model for the endogenous variables.

**Table 3:** The results of path analysis.

Pathways	Unstd	S. E.	C. R.	*p*	Std	R^2^
PP → IOU	−0.322	0.054	−5.982	***	−0.301	0.091
IOU → CDD	0.463	0.05	9.174	***	0.471	0.311
PP → CDD	−0.197	0.049	−3.99	***	−0.188	
CDD → CCA	0.334	0.049	6.847	***	0.342	0.589
PP → CCA	−0.177	0.043	−4.162	***	−0.173	
IOU → CCA	0.424	0.049	8.579	***	0.443	

The symbol *** presented in Table 3 indicates that the *p*-value is less than 0.001 (*p* < 0.001), meaning that the results are highly statistically significant.

**Figure 2:**
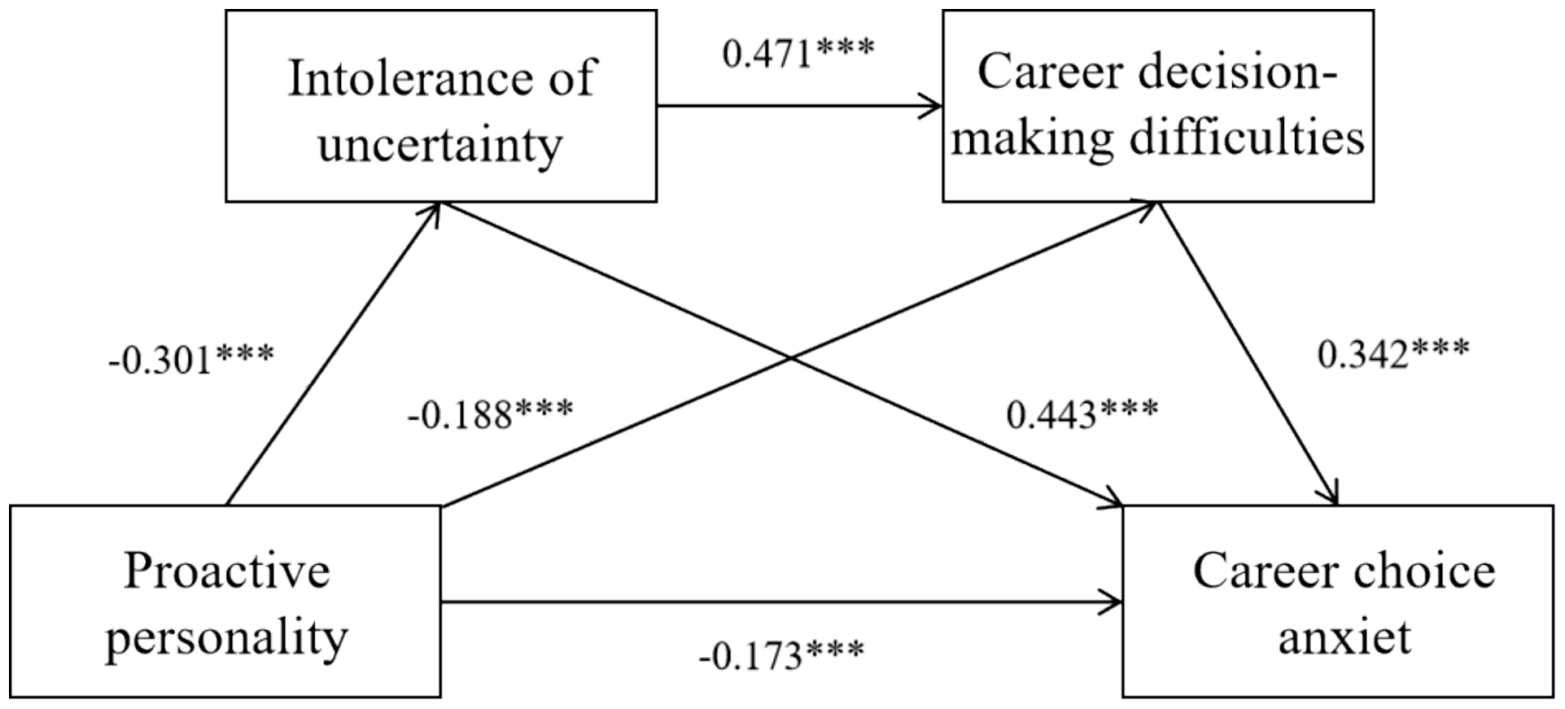
The standardized path of college students’ proactive personality and their career anxiety.

The bias-corrected percentile Bootstrap method was employed to test the mediating effects, with the number of samples set at 5000. The specific effect values and their 95% confidence intervals are detailed in Table 4 (Yuan et al., 2024). The results indicated that the total effect of proactive personality on career choice anxiety was −0.371 (95% CI: [−0.4527, −0.2897]), indicating a significant link where higher levels of proactive personality correspond with lower levels of career choice anxiety, thereby further supporting Hypothesis 1. After controlling for the mediating variables, the direct effect of proactive personality on career choice anxiety remained significant, with an effect value of −0.177 (95% CI: [−0.2455, −0.1081]), indicating the presence of partial mediation. First, the indirect effect of the path “proactive personality → intolerance of uncertainty → career choice anxiety” was estimated at −0.099 (SE = 0.019), and its 95% confidence interval [−0.1375, −0.0637] did not include zero. This confirmed the partial mediating role of intolerance of uncertainty, suggesting a pathway where individuals with lower proactive personality tend to exhibit higher intolerance of uncertainty, which is linked to higher career choice anxiety. Second, the indirect effect of the path “proactive personality → career decision-making difficulties → career choice anxiety” was calculated as −0.062 (SE = 0.014), and its 95% confidence interval [−0.0904, −0.0355] also excluded zero, indicating that career decision-making difficulties played a significant mediating role in this relationship. Hypotheses 2 and 3 were further supported. Finally, the third path, representing the Chain mediation of “proactive personality → intolerance of uncertainty → career decision-making difficulties → career choice anxiety,” was also found to have a negative indirect effect value of −0.034 (95% CI: [−0.0486, −0.0208]), thereby validating Hypothesis 4. In conclusion, proactive personality among university students was found to not only directly influence their career choice anxiety but also indirectly affect it through the mediating roles of intolerance of uncertainty and career decision-making difficulties (Figure 2).

**Table 4:** Results of Bootstrap mediated effects analysis.

Impact pathways	Effect	BootSE	BootLLCI	BootULCI	Effect size ratio
Total effect	−0.371	0.042	−0.4527	−0.2897	
Direct effect	−0.177	0.035	−0.2455	−0.1081	47.71%
PP → IOU → CCA	−0.099	0.019	−0.1375	−0.0637	26.68%
PP → CDD → CCA	−0.062	0.014	−0.0904	−0.0355	16.71%
PP → IOU → CDD → CCA	−0.034	0.007	−0.0486	−0.0208	9.16%

## 4 Discussion

Based on the theoretical frameworks of Social Cognitive Career Theory and Cognitive-Behavioral Theory, this study constructed a chain mediation model to systematically examine the mechanism through which proactive personality influences career choice anxiety among university students. The results supported all hypotheses: proactive personality was found not only to directly and negatively predict career choice anxiety, but also to exert indirect effects through the independent mediating roles of intolerance of uncertainty and career decision-making difficulties, as well as their sequential mediating pathway. These findings provide a novel theoretical perspective for understanding the formation mechanism of career choice anxiety and deepen our comprehension of how personality traits influence emotional states through cognitive-behavioral pathways.

First, this study confirms proactive personality demonstrated a significant direct negative association with career choice anxiety among university students (Song et al., 2025), consistent with its theorized role as a mitigating factor in career-related stress. When facing career choices and employment challenges, individuals characterized by high levels of proactive personality typically exhibit stronger goal-oriented behaviors, enhanced forward-planning capabilities, and more proactive problem-solving strategies (Pan et al., 2018). According to Social Cognitive Career Theory, personality traits profoundly influence emotional outcomes by shaping individuals’ cognitive appraisal frameworks and behavioral response patterns. Students with high proactive traits are more inclined to actively build extensive social support networks, acquire career information and practical opportunities, and demonstrate higher career decision-making self-efficacy when confronting uncertainty (Ugwu et al., 2021). These positive cognitive and behavioral strategies can theoretically alleviate anxiety stemming from information asymmetry, ambiguous career goals, or employment competition pressure. Particularly against the backdrop of increasingly complex global employment situations and diversified career paths, the psychological protective function provided by proactive personality is deemed especially crucial (Zhao et al., 2022). This result not only reinforces the theoretical value of proactive personality as a positive psychological trait but also provides empirical support for incorporating proactive personality development into the mental health education and career guidance systems within higher education institutions, pointing the way for designing prevention and intervention strategies addressing career choice anxiety among university students.

Second, the study found that intolerance of uncertainty mediates the relationship between proactive personality and career choice anxiety, aligning with the core proposition of the cognitive risk appraisal model, which emphasizes the crucial role of cognitive appraisal of uncertain situations in emotional regulation processes. The results indicate that a higher level of proactive personality is associated with greater tolerance for uncertainty, which in turn is linked to lower anxiety during the career decision-making process (Furnham et al., 2025). Regarding the underlying mechanism, highly proactive individuals generally possess greater cognitive flexibility, enabling them to reappraise ambiguous situations in career choices as potential opportunities for personal growth and development rather than as threatening stimuli (Zhang and Zheng, 2025). This positive cognitive restructuring strategy helps individuals maintain a calmer and more rational attitude when confronting career-related uncertainties (Arbona et al., 2021). This discovery not only deepens our understanding of the mechanism through which personality traits influence emotional responses via cognitive pathways but also provides a theoretical basis and practical entry point for future psychological interventions based on cognitive-behavioral strategies, suggesting that we can indirectly alleviate career anxiety by enhancing individuals’ cognitive flexibility and tolerance for uncertainty.

Third, career decision-making difficulties were confirmed to play an independent mediating role between proactive personality and career choice anxiety. The findings demonstrate that proactive personality can directly and negatively predict career choice anxiety, and can also indirectly reduce anxiety levels by alleviating career decision-making difficulties (Chen and Zhang, 2024). Individuals with high levels of proactive personality are more inclined to engage in active career exploration, proactively acquire career-related information, and continuously clarify their self-concept (Ruan et al., 2025). These proactive career construction behaviors help mitigate decision-making difficulties caused by insufficient information, ambiguous career goals, or internal conflicts, thereby significantly reducing anxiety during the career selection process (Le et al., 2024). This finding resonates with the core proposition of career construction theory, which posits that through active career adaptation behaviors, individuals are better equipped to cope with challenges in the vocational environment, consequently enhancing decision-making efficacy and psychological adaptation (Merino-Tejedor et al., 2025). The mediating role of career decision-making difficulties, a proximal influencing factor of career choice anxiety, highlights the important practical implications of enhancing students’ career decision-making readiness and competencies within career development guidance.

Most importantly, this study further revealed a chain mediation pathway through intolerance of uncertainty and career decision-making difficulties. This pathway delineates a complete mechanism of influence progressing from “cognitive appraisal” to “behavioral decision” and ultimately to “emotional outcome.” The model indicates that intolerance of uncertainty plays a critical psychological transmission role in the career decision-making process: it not only acts as a cognitive bridge through which personality traits influence decision-making behaviors but also, by inducing decision avoidance tendencies, exacerbates difficulties encountered during career exploration, which are ultimately translated into persistent anxiety (Du et al., 2024). This pathway clearly demonstrates that career choice anxiety is not merely an emotional response but rather the emotional consequence of a series of passive behavioral patterns triggered by cognitive appraisal biases when confronting career uncertainties (Wang et al., 2017). From the perspective of cognitive-behavioral interaction, this finding deepens our understanding of the formation process of career decision-making difficulties and provides a theoretical entry point for targeted career psychological interventions.

In summary, by integrating social-cognitive and cognitive-behavioral perspectives, this study systematically reveals the multiple pathways through which proactive personality influences career choice anxiety. The results emphasize the need for a comprehensive intervention approach to alleviate career anxiety among university students: it is essential to focus on cultivating and stimulating positive traits like proactive personality, while also intervening in the underlying cognitive appraisal processes and specific behavioral manifestations. The conclusions of this study provide a complete theoretical framework and practical guidance for higher education institutions to construct a career psychological service system integrating “trait cultivation, cognitive adjustment, behavioral support, and emotional guidance.”

## 5 Implications and limitations

Through the construction of a Chain mediation model, the mechanism through which proactive personality influences career choice anxiety was systematically elucidated in this study, with intolerance of uncertainty and career decision-making difficulties confirmed as playing key mediating roles. Theoretically, this study moves beyond simple replication of known correlations by explicitly integrating cognitive appraisal (intolerance of uncertainty) and behavioral decision-making (career decision-making difficulties) into the Social Cognitive Career Theory (SCCT) framework. While SCCT acknowledges the role of person inputs and cognitive processes, it often treats cognitive variables like self-efficacy in a more generalized manner. By specifying intolerance of uncertainty—a specific cognitive bias in the face of ambiguity—as a critical proximal cognitive mechanism, and by delineating its sequential influence on concrete career decision-making behaviors (difficulties) and then emotional outcomes (anxiety), this model provides a more granular and context-specific pathway within SCCT. It explicates how a broad personality trait (proactive personality) translates into a specific career-related emotional outcome by unpacking the intermediary cognitive and behavioral steps, thereby extending SCCT’s explanatory power for understanding career distress in highly uncertain environments. This clearly delineates the complete pathway of “personality trait → cognitive bias → behavioral difficulty → emotional outcome.” This not only deepens the application of Social Cognitive Career Theory in explaining the formation mechanism of career choice anxiety but also broadens the understanding of how personality traits influence emotional outcomes from a cognitive-behavioral linkage perspective. The validated Chain mediation mechanism indicates that career choice anxiety is not directly caused by a single factor but is progressively formed through the sequential transmission of cognitive and behavioral factors, providing a more refined theoretical explanation for understanding university students’ psychological adaptation in employment contexts.

At the practical level, this study offers direction and intervention pathways for career guidance and psychological health education in higher education. First, proactive personality should be cultivated through career planning courses, role model demonstrations, and practical projects to stimulate students’ tendency for active exploration and planning. Second, targeted cognitive flexibility training should be implemented, utilizing cognitive-behavioral therapy, mindfulness practices, and uncertainty scenario simulations to enhance students’ tolerance for career-related ambiguity and reframe their cognitive appraisal of employment pressure. Furthermore, systematic support for the career decision-making process should be strengthened by providing services such as occupational information integration, decision-making skills training, and goal clarification workshops to reduce difficulties in career choice. Ultimately, informed by the identified mechanisms, a comprehensive four-in-one career psychological service system—encompassing “trait cultivation, cognitive adjustment, behavioral support, and emotional guidance”—can be established in higher education institutions. This system would achieve full-process coverage, alleviate students’ career choice anxiety, and facilitate rational employment and positive development.

Despite the rigorous approach adopted in this study, several limitations should be noted, which point to directions for improvement and further investigation. First, regarding the research design, cross-sectional data were utilized. Although such data can effectively reveal correlations and mediating pathways among variables, they do not allow causal relationships to be established. Complex bidirectional influences or dynamic interactions may exist among career choice anxiety, intolerance of uncertainty, and career decision-making difficulties. Future studies could employ longitudinal tracking designs or experimental intervention methods, with variables measured at multiple time points, to more accurately elucidate causal sequences and long-term effects. Second, in terms of sampling and methodology, the sample was drawn from universities in a single province and recruited through convenience sampling. The uneven distribution of gender, academic year, and institution type may limit the generalizability of the findings. Moreover, although common method bias was partially controlled procedurally, data were primarily collected through self-report questionnaires, and the potential influence of social desirability or recall bias cannot be entirely ruled out. Future research could expand the sampling scope to include student populations from diverse regions and institutional tiers, and incorporate multi-source data such as behavioral observations, interviews, and objective indicators to enhance the reliability and external validity of the results. Furthermore, concerning the theoretical contribution and variable operationalization, while the “cognition-behavior-emotion” pathway is indeed a core principle of cognitive-behavioral theory, the specific contribution of this study lies in empirically testing and validating this sequence within the specific domain of career decision-making, linking a broad personality trait to a specific anxiety outcome via theoretically-specified and domain-relevant mediators (intolerance of uncertainty and career decision-making difficulties). Furthermore, with regard to variable selection and mechanism exploration, this study focused on cognitive and behavioral mediating pathways. However, the development of career choice anxiety may also be influenced by multi-level factors such as social support, family financial pressure, cultural values, and the macro employment environment. Future research could introduce variables such as environmental factors, psychological capital, and digital literacy to examine their moderating roles or conditional indirect effects, thereby constructing a more comprehensive and context-specific theoretical model to further reveal the formation mechanisms and intervention pathways of career choice anxiety among university students.

## 6 Conclusion

A Chain mediation model was constructed to systematically examine the mechanism through which proactive personality influences career choice anxiety among university students. The independent mediating roles of intolerance of uncertainty and career decision-making difficulties, as well as their sequential mediating pathway, were empirically identified. The findings revealed that proactive personality was not only directly and negatively associated with career choice anxiety, but its higher levels were also linked to lower intolerance of uncertainty and reduced career decision-making difficulties, which in turn corresponded with lower anxiety. This “cognition-behavior-emotion” pathway deepens the understanding of the formation mechanism of career choice anxiety from an integrated social-cognitive and cognitive-behavioral perspective, and provides a solid theoretical foundation for understanding the relationships between these variables along with clear practical directions to inform career psychological guidance in higher education institutions. Future research would be recommended to further verify causal relationships among variables through longitudinal tracking or experimental interventions, expand the scope of sampling, and incorporate multi-level influencing factors, thereby enabling the construction of a more systematic model of vocational psychological adaptation among university students.

## Data Availability

The raw data supporting the conclusions of this article will be made available by the authors, without undue reservation.
